# The Role of Nurses in Primary Care: Managing Alcohol-Abusing Patients

**Published:** 1994

**Authors:** Eleanor J. Sullivan, Sandra M. Handley, Helen Connors

**Affiliations:** Eleanor J. Sullivan, Ph.D., R.N., F.A.A.N., is the dean and a professor; Sandra M. Handley, Ph.D., R.N., C.A.R.N., is a research associate; and Helen Connors, Ph.D., R.N., is an associate dean and associate professor at the University of Kansas School of Nursing, Kansas City, Kansas

## Abstract

Nurses work on the front lines of primary health care delivery in many settings. The unique characteristics of nursing care put nurses in an excellent position to identify, assess, counsel, and monitor clients with alcohol problems.

Primary health care is more than community-based health care or primary medical care. It is “the first level of contact of individuals with the health system bringing health care as close as possible to where people live and work, and constitutes the first element of a continuing health care process” (World Health Organization 1988, p. 21). Thus, primary health care includes health promotion, disease prevention and treatment, and rehabilitation services. Nurses are an integral component of the primary health care system. Working in a variety of settings, they are front-line providers of these services.

Alcohol abuse and dependence and their associated medical, psychological, and social consequences are encountered in all areas of primary health care. It is estimated that 18 to 20 percent of patients in ambulatory settings have alcohol abuse or dependence problems (Searight 1992). Between 30 and 50 percent of all hospital admissions also are related to the effects of alcohol abuse (Bush et al. 1987).

In many ways, nurses’ roles in prevention, detection, treatment, and referral of patients with alcohol problems are similar to the roles of other health care professionals. These generic roles include screening clients with appropriate instruments; following up on patients who have positive screening results; and providing assessment, intervention, and referral. Nurses also should develop a repertoire of resources to help them determine the best treatment alternatives for each patient. Such resources include a certified addictions nurse or other substance abuse expert, local chapters of Alcoholics Anonymous, State agencies for alcohol and drug abuse, and the National Council on Alcohol and Drug Dependence (see the resources guide in Barry and Fleming, p. 108).

However, many unique qualities of nursing practice make nurses particularly well suited to assist alcohol-abusing clients. In this article, we review these characteristics. We describe some of the primary care settings in which nurses work and the types of alcohol-related problems and intervention approaches they encounter. We also present factors that influence nurses’ attitudes toward patients with alcohol disorders and how, in turn, they affect nurses’ abilities to help those patients adequately.

## Unique Aspects of Nursing

### A Holistic Approach

Nursing practice gives equal importance to the physiological, psychological, and socio-cultural characteristics of the client (Kozier et al. 1991). “Holistic” means that all these aspects are assessed routinely by the nurse and integrated into a picture of the client that is more complete than one based solely on the symptoms of the immediate medical complaint. Information on the patient’s combined characteristics forms the basis of a statement of client problems—the nursing diagnosis (Potter and Perry 1993).

Such a holistic approach is important particularly for patients with alcohol-related problems; alcohol’s contribution to an illness may remain obscure if the nurse focuses only on the clinical symptoms. For example, a medical complaint, such as gastrointestinal upset, along with psychological data related to stress and socio-cultural information related to a family history of alcohol abuse all may be necessary to reveal to the nurse that a patient has an underlying alcohol problem.

As part of the holistic treatment approach, nursing focuses not only on the illness but also on the patient’s, response to the illness. For example, a client’s denial of alcohol abuse may be the immediate problem: the client must acknowledge the problem before it can be treated successfully.

### Individualized Patient Care

Nursing diagnosis and care are tailored to fit the needs of the patient (Potter and Perry 1993). Using this approach, care can be continually updated based on the client’s progress and experiences. For example, the level of family involvement in treatment may have to be revised if the family situation changes. The ability to deliver individualized care is useful especially for alcohol-abusing patients because they often vary considerably in their abuse patterns, personal characteristics, and family situations.

### Collaborative Patient Care

The nursing process relies on a strong partnership between the nurse and the patient. Problems should be addressed by both parties, not by the nurse alone (Carson 1992). By including the patient in both problem definition and problemsolving, the nurse ensures that the patient’s personal situation, perspective, and current level of understanding of the disease are taken into consideration. Collaboration also encourages patients to participate in their care and take responsibility for their recovery.

### Involving the Client’s Family

Because alcohol abuse may both result from and affect the patient’s family situation, it is important to address problems related to this environment. Nurses often have contact with the client’s family, and they are trained to analyze family dynamics and to identify patient-family interactions that may help or hinder recovery. This information may be shared with the client or family to increase their understanding of the family dynamics. Such skills also are crucial for providing care to the family and for involving family members in the client’s care.

### Emphasizing Health Education

Nurses routinely assess their patient’s knowledge about health and illness and provide information targeted to the client’s level of understanding (Murray and Zentner 1993). Thus, they are in an excellent position to teach clients and their families about (1) responsible alcohol use; (2) risk factors, such as a family history of alcohol abuse or excessive use connected with work or recreation; (3) alternatives to alcohol use and abuse (e.g., stress management and recreational activities); and (4) the disease of alcoholism. For recovering clients, education can focus on how to maintain and stabilize recovery, teaching them stress management and relapse prevention techniques.

### Reliance on Interpersonal Skills

The nurse relies on strong interpersonal and interviewing skills to establish a working relationship with the client and improve the chances of correctly identifying and treating a problem (Arnold and Boggs 1989). These skills are important particularly when dealing with alcohol-abusing clients, who may be unwilling to disclose information about their use of alcohol.

### Making Care Accessible

Nurses work in various environments and frequently are the first contact the patient has with the health care system. Nurses often are more accessible and establish longer, more in-depth relationships with the clients and their families than do other health care providers. Consequently, patients may regard nurses as informal, nonthreatening sources for obtaining information on alcohol or other drug problems.

## Specific Nursing Settings

Although all nursing care is based on the characteristics discussed above, some settings may require more specialized skills, depending on the type of clients involved.

### Hospitals

Many nurses work in hospitals where care often focuses on the physical consequences of alcohol abuse and on identifying and managing patients with alcohol withdrawal symptoms (Marcus et al. in press). The nurse’s interpersonal and observational skills especially are useful in this setting to identify and address the underlying alcohol problems. A hospital setting also provides many opportunities for educating clients and their families about alcohol abuse and its medical consequences.

### Schools

Nurses working in elementary or middle schools encounter students from alcohol-abusing families as well as students experimenting with alcohol or other drugs. Children of alcoholics often come to the attention of the school nurse because of frequent physical, or nonspecific, complaints, or they may be referred to the nurse by a teacher (Shenk in press). The nurse then may refer these students to an existing program for children of alcoholics or develop such a program. The nurse also serves as a source of information, assessment, or referral for treatment for students who are experimenting with or abusing alcohol.

The nurse often is the first to contact the parents of children who are having problems (directly or indirectly) with alcohol. Such contact allows the nurse to evaluate the family situation and to share information on the student’s problem. In addition to providing immediate health services, school nurses often are involved with school-based alcohol and other drug primary prevention programs.

Secondary schools frequently employ nurse practitioners who identify and assess students with alcohol problems and refer them to alcohol abuse specialists. In other secondary school settings, however, the substance-abuse prevention programs are managed by the students. In such instances, nurses may have only a supportive role in prevention and education.

### Workplaces

Occupational health nurses (OHN’s) work independently or through employee assistance programs (EAP’s). The OHN’s role mainly entails developing prevention programs and identifying employees with alcohol problems (e.g., during routine physical examinations or because of an injury or incident in the workplace). Employees with alcohol problems can be referred for treatment to the EAP or to outside programs. When recovering employees return to work, the OHN may be involved in posttreatment monitoring (e.g., performing random drug tests) (Shenk in press).

### Homes

Home health nurses deliver health care to homebound clients. This is an excellent opportunity to assess the patient’s family, environment, and social functioning. Home visits also allow the nurse to follow up on a client or family member who was referred to treatment but who declined to comply. Such visits provide the nurse with another opportunity to intervene and refer. For recovering clients, the nurse may help implement and reinforce recommended lifestyle changes and monitor for relapse. The home health nurse also sees many elderly patients who may be at increased risk for alcohol abuse because of age-related impairments or social isolation (Shenk in press).

### Clinics, Nursing Clinics, and Medical Offices

Nurses working in clinic and office settings provide all aspects of care for patients with alcohol problems, including client education, screening, and referral if needed (Shenk in press).

### Emergency Departments

The nurses role in an emergency department often includes assessing the extent of involvement of alcohol or other drugs in the emergency. Therefore, nurses need accurate knowledge of the effects, side effects, and toxicity of a variety of abused substances (Marcus et al. in press). Patients in emergency rooms typically are under stress and are emotionally vulnerable. Thus, they may be particularly receptive to referrals by the nurse for further evaluation of their alcohol problems, especially if the nurse can follow up on the referral.

## Factors That Affect Care

The ability of nurses (as with other health care professionals) to identify and address alcohol abuse in clients is affected by many factors. These include the nurses’ level of education; their beliefs and attitudes, which are shaped by personal and professional experiences; and the commitment of their health care organization to the detection of and care for clients with alcohol problems.

### Nursing Education

Without the appropriate knowledge, nurses are poorly prepared for the complex issues that surround the care of patients with alcohol problems. They may be unable to deal with clients’ denial (Bartek et al. 1988), or they may set unrealistic goals for these patients. For example, whereas a nurse may want a client to respond immediately to information about the effects of excessive alcohol use, a more appropriate goal might be to plant the idea in the client’s mind that drinking may be harmful.

Several surveys of curricula have revealed a paucity of substance abuse education in nursing schools today. For instance, a national survey of 335 nursing schools found that 72 percent devoted less than 5 class hours to the topic of alcohol and other drug abuse (Hoffman and Heinemann 1987).

The Federal Government, private foundations, and selected nursing schools have initiated efforts to correct this educational deficit. With funding from the Center for Substance Abuse Prevention (CSAP), the National Institute on Alcohol Abuse and Alcoholism and the National Institute on Drug Abuse recently developed three model curricula on alcohol and other drug abuse for nursing schools. These curricula are now available and include lesson plans, overhead transparencies, and bibliographic references (Naegle 1991–1993; Church et al. 1992; Burns and Thompson 1993; for more information, see the article by Naegle, pp. 154–157).

In addition to the curriculum models, CSAP also has funded a faculty development initiative to increase faculty expertise in alcohol and other drug abuse in nursing and medical schools, psychology departments, and social work programs. Faculty members are trained to integrate substance abuse education throughout the nursing curriculum instead of presenting the material only as part of general mental health education.

Preliminary results of these initiatives are encouraging. For example, Handley and colleagues (1993) reported that after a series of 2-day faculty development workshops, 77 percent of the participants reported using the information from the workshops in their classes.

In addition to these curricula initiatives, CSAP funding has led to the development of many local projects (e.g., workshops). Also, nursing and medical faculty experts have contributed to a textbook, *Nursing Care of the Client with Substance Abuse*, for use in all clinical areas (Sullivan in press).

### The Effect of Attitude on Care

Nurses’ perceptions of alcohol abuse have an effect on the amount and quality of care they provide to patients with these problems. Their attitudes are shaped both by the knowledge (or lack thereof) about alcohol abuse and by previous personal and professional experiences.

Studies on nurses’ attitudes toward alcohol and other drug abuse have been contradictory. On a professional level, nurses may believe that alcohol abuse is caused by psychological or physical-genetic factors rather than lack of will-power. However, some studies have found that on a personal level, nurses generally are less permissive than other health care professionals regarding alcohol and other drug use in society, and they have more negative attitudes toward substance-abusing clients (for a review of these studies, see Sullivan and Handley 1993). These findings suggest that although in theory nurses do not blame alcohol-abusing patients for their illness, in practice they may find it difficult to care for and understand these clients.

Nurses’ attitudes toward substance-abusing patients can be affected positively by educational and clinical experiences (Jack 1989). Therefore, recently developed curriculum models (see above) include modules to help students identify and address their attitudes toward these clients. A nursing curriculum on substance abuse prevention also includes a component on attitudes (National Nurses Society on Addictions [NNSA] and American Nurses Association [ANA] 1992). These components emphasize approaches to counteracting the perception that alcoholism treatment is futile. Still, the strongest impact on attitudes may result from listening to recovering alcoholic patients speak about their experiences, thus putting a face on the disease.

#### Personal Experiences

Like many people, nurses are affected by alcohol abuse in their own family members or friends. Sullivan (1987) found that more than 25 percent of randomly selected nurses reported having an alcoholic family member. Also, two-thirds of nurses identified as having an alcohol or other drug problem also had an alcoholic family member.

The extent to which nurses’ experiences with their own alcohol-abusing family members influence their professional practice depends on the magnitude of the family problems and the degree to which they have been resolved. For some people, the experience of growing up in families with alcohol or other drug abuse may contribute to choosing a career in nursing. Children in these families often are caretakers for other family members, a role that may be carried over into adult life in the form of a nursing or other health care career (Black 1982; Sullivan et al. 1988).

Nurses who have resolved problematic experiences with alcohol or other drug abuse in their families can be excellent resources for their clients. Reisman and Shrader (1984) found that OHN’s with knowledge and personal experience with alcohol abuse were more likely to refer clients appropriately. On the other hand, unresolved family issues associated with alcohol and other drug abuse may make it difficult for a nurse to address objectively such problems in patients (NNSA and ANA 1992).

Consistent with data for the general population, 3 to 4 percent of nurses have a current or past substance abuse problem (Trinkoff et al. 1991; Sullivan 1987). If they are heavy users or abusers of alcohol or other drugs themselves, they are unlikely to be able to address clients’ problems. Nurses recovering from alcoholism, however, may be uniquely able to provide empathetic care and advice to those with alcohol problems.

#### Professional Experiences

Nurses often see patients with the most dramatic consequences of both acute and chronic alcohol abuse. Many individuals repeatedly require treatment for an alcohol-related illness or relapse after alcoholism treatment. Because of this experience, nurses may consider unsuccessful recovery as the norm, causing them to have negative attitudes toward all alcohol-abusing patients (Zahourek 1986).

To develop a better understanding of alcohol-related problems, nurses have to be aware that alcoholism is a chronic disease characterized by loss of control and the potential for relapse. As with all chronic diseases, treatment can be frustrating to professionals in a high-tech, cure-oriented health care system (Kinney et al. 1984), especially because there are few clearly defined treatment protocols and demonstrated outcomes. Nurses should realize that their clients often represent behavioral extremes rather than the norm. Attending a 12-step group as a professional visitor may help them to better understand the full range of treatment outcomes that are possible.

### Role of Health Care Organizations

Most primary health care is delivered in the context of defined organizations, such as hospitals, clinics, schools, companies, or physicians’ offices. The policies of these organizations, both formal and informal, greatly affect the nurse’s ability to detect and respond to patients who abuse alcohol.

Although almost all health assessments include questions on alcohol use, these questions may be deferred routinely or the information may not lead to appropriate intervention (Tweed 1989). In addition, nurses may be uncertain about identifying alcohol abuse in clients with other medical complaints. The efficiency of alcohol abuse detection could be increased if all health care providers screened clients for substance abuse and responded to the results in a similar way. Such standards can be promoted through educational presentations, group meetings, and development of unambiguous policies within organizations or at professional meetings of multiple organizations.

After identifying an alcohol problem, the nursing staff must be prepared to respond appropriately. A substance abuse specialist in the organization can provide guidance about nursing interventions and referral sources. This expert can be an addictions nurse, a mental health specialist, or a nurse with the interest or experience to develop the expertise (Leiker 1989).

## Summary

Nurses who are knowledgeable and skilled in education, assessment, prevention techniques, risk factor identification, and counseling are well equipped to care for patients with alcohol-related problems. Nurses often have regular contacts with their clients, enabling them to identify high-risk individuals and educate them about alcohol abuse. In addition, nurses’ contributions to early detection and intervention can reduce the effects of long-term, excessive drinking on the individual, the family, and society.

Yet until now, nurses’ efficiency in dealing with alcohol-related problems has been hampered occasionally by insufficient substance abuse education. Because alcohol and other drug abuse occurs in conjunction with several other diagnoses and in all clinical areas, substance abuse education should be integrated throughout the nursing curriculum rather than treated only as a mental health problem.

Substance abuse as a subspecialty in nursing draws clinicians from diverse backgrounds. All nursing students should receive at least a minimum level of substance abuse education to continue to elicit interest in this specialty. The current emphasis on primary care makes this an ideal time to improve nursing care for alcohol-abusing clients by developing better professional education, addressing negative attitudes toward these clients, and increasing organizational commitment to the treatment of alcohol disorders. By capitalizing on the unique qualifications of nurses, timely and cost-effective care delivery to clients with alcohol-related problems could be ensured.
